# Supplementary material for: Are carbon emissions trading and green financial instruments synergistic? -Comprehensive quantitative research based on content analysis
Source: PLoS One. 2024 Mar 7;19(3):e0298601. doi: 10.1371/journal.pone.0298601 (PMC10919601; doi:10.1371/journal.pone.0298601)
Supplement: S1 Data — (ZIP) [file pone.0298601.s001.zip › Data/地级市Z_F工作报告.zip!/║■▒▒/╢⌡╓▌╩╨/└┤╫╘╣1⁄2╓┌║┼í╛┬φ┐╦╩2╛▌═°í┐_2003.docx]

各位代表：

　　我代表市人民政府向大会作政府工作报告，请予审议，并请市政协各位委员和列席人员提出意见。

　　一、2002年工作回顾

　　2002年是我市建设富裕型小康城市的起步年。一年来，全市人民在省委、省政府和市委的正确领导下，高举邓小平理论伟大旗帜，认真实践“三个代表”重要思想，团结一心，克难勇进，扎实工作，圆满完成了市四届人大第四次会议提出的各项任务。

　　 ——经济建设迈上新台阶。全年实现国内生产总值113.2亿元，比上年增长10.6%；城镇居民人均可支配收入6404元，农民人均纯收入2693元，分别增长8.7%和3.9%。工业经济持续增长。全市规模以上工业企业完成工业增加值38.22亿元，增长12.1%。高新技术产业发展较快，全年完成高新技术产业增加值7.48亿元，增长24.9%。农业支柱产业逐步形成。全年完成农业增加值15.8亿元，同比增长3%。农业结构进一步优化，水产业产值占农业总产值的比重达40.98%，首次超过种植业。农田水利基本建设取得新进展，全年完成水利建设投资1.85亿元。退耕还林迈出新步伐，全年完成造林面积7.6万亩。财政金融运行平稳。全年完成财政收入7.54亿元，增长10.6%。收入结构趋于优化，国、地两税收入占财政收入的比重达89%，同比提高14.2个百分点。金融机构年末各项存款余额73.39亿元，各项贷款余额63.22亿元，较年初分别增加12.37亿元和5.4亿元。信用环境建设取得实质性进展。整顿和规范市场经济秩序工作卓有成效，城乡市场繁荣，社会消费品零售总额完成44.92亿元，增长9.6%。项目建设进展顺利。全年完成固定资产投资32.28亿元，增长11%。年初安排的56个重点项目，建成投产14个，开工建设19个，其它项目正抓紧进行前期工作。项目科技含量不断提高，一年来共实施国家和省级科技项目53项，获省科技进步奖5项。

——改革开放取得新突破。国企“两改”扎实推进。全市重点企业已完成“两改”104家，协议安置职工3.85万人。债务处置和资产变现工作力度加大，与信达公司达成债务清偿协议，72家企业呆滞贷款得到妥善处置。多佳、武昌鱼两家上市公司顺利实现资产重组，双双成为绩优股。全年新发展个体私营企业4047家，增长21.3%。农村税费改革基本完成。全市315个村税费改革方案全部落实到户，签字率达95%以上。税改后，全市农民负担总额减少4200万元，减负率达49%，人平减负58.06元，亩平减负64.04元，现人平负担为60.75元，亩平负担为84.2元。行政管理体制改革逐步深化。成立了经济发展联合办公中心，首批22个部门213个审批项目、114个收费项目进中心办理。继党政机构改革之后，全市事业单位改革精简机构37.9%，精减编制21.5%，事业单位合同签订率、鉴证率均达到98%。财会集中核算、部门预算、政府采购等财政综合改革成效明显，财政收支结构得到优化。国土资源管理迈出新步伐，经营性土地全部实现市场挂牌、拍卖交易。对外开放成效显著。全年组织参加港澳招商会、深圳高交会等大型招商活动6次，引进落实武钢球团矿厂、红莲湖旅游开发等投资500万元以上项目52项，完成招商引资额1.35亿美元(其中外商直接投资4523万美元)，同比增长12.5%，新批外资企业12家，增长50%。全年外贸出口总额17.31亿元(其中出口海关数5365万美元)，同比增长9%。葛店高新技术开发区招商引资工作取得新进展，全年新批注册项目13个，在建项目16个。

——城市面貌发生新变化。以推进城镇化建设为着力点，创建中国优秀旅游城和省级文明城、卫生城、山水园林城工作进展顺利。城市基础设施建设步伐加快。全年共完成城市基础设施建设投资4.55亿元，同比增长13.2%。凤凰广场、鄂黄大桥、鄂州高中等标志性建设工程为城市增添了新的亮点。小城镇和中心村建设保持良好发展态势，城镇化水平比上年提高4.5个百分点。城乡电网、通讯网、路网建设取得新成效，新建农村三、四级水泥路面244公里，使85%的村通了水泥路。樊湖线完成改扩建任务，鄂黄大桥连接线建成通车。生态环境整治力度加大。洋澜湖治理和生态保护进一步加强，清淤工程基本完成。大力实施城市净化、绿化、亮化、美化工程，顺利进入省级园林城市行列。旅游功能进一步提升。全年投入旅游设施建设资金1.7亿元，景区、景点建设步伐加快。运用市场手段成功举办“武昌鱼国际文化旅游节”和“梁子湖捕鱼旅游节”，全年接待国内外游客115.3万人次，实现旅游收入7.87亿元，分别增长16.7%和17.6%，通过了国家优秀旅游城市验收。

——社会事业取得新进展。全面贯彻实施《公民道德建设实施纲要》，广泛开展“道德天天讲、文明伴我行”的主题教育活动，群众性精神文明创建工作深入进行，市民素质进一步提高，我市再次夺得省级“文明城市”光荣称号。教育改革取得显著成果，教育资源不断优化，中小学校布局逐步调整到位。文化体育事业进一步繁荣。卫生事业长足发展，科技、文化、卫生“三下乡”活动成效明显。“双拥”工作扎实开展，我市被省委、省政府、省军区命名为“双拥模范城”。社会保障体系进一步完善，“两个确保”得到巩固。再就业工作力度加大，城镇登记失业率为3.5%。计划生育率同比提高0.3个百分点，稳定了低生育水平，全市人口自然增长率为3.75‰。社会治安综合治理与严打整治工作取得成效，保持了全市城乡社会持续稳定。“四五”普法开局良好，民主法制建设不断加强。基层民主政治建设稳步推进，圆满完成村级两委换届选举。深化以“制权、管钱、用人”为重点的三项制度改革，大案要案查处工作力度加大，源头治腐取得明显成效。审计、对台、外事侨务、档案、民族宗教、广播电视等各项工作取得新成绩，人防、气象、信访、扶贫、老龄、残疾人保障等事业有新发展。去年，市政府承诺为市民办的8件实事，城镇居民最低生活保障应保尽保、建成150公里村级水泥路、修建凤凰广场、鄂州高中主体工程建设等4件提前或超额完成；洋澜湖生态治理，建设武昌鱼桥，城市地名设标，乡乡镇镇通自来水、村村户户饮用卫生水等4件均完成年度计划，余下工作在2003年完成。各位代表，过去的一年，我市各项工作取得的成绩来之不易，凝聚着全市人民的智慧和艰辛。借此机会，我代表市人民政府向全体代表、政协委员，向为我市经济社会发展作出贡献的广大干部群众、知识分子、驻军指战员、武警官兵、公安干警、离退休老同志和各民主党派、工商联、人民团体、无党派人士致以崇高的敬意！向所有关心支持我市建设的港澳台同胞、海外侨胞、外国友人表示衷心的感谢！在充分肯定成绩的同时，我们也清醒地看到，当前全市经济社会生活中仍然存在一些困难和问题，主要是：经济发展不平衡，区乡财政增长乏力；产业结构不尽合理，企业市场竞争力不强；农业产业结构调整任务艰巨，农民增收缓慢；下岗职工增多，再就业形势严峻。这些困难和问题有的是长期积累下来的，有的是体制转型和结构调整过程中的“阵痛”，有的是由于我们的思维方式、工作方式乃至工作作风与新形势、新任务的要求不相适应造成的。在今后的工作中，我们必须正视这些问题，采取得力措施，认真加以解决。

二、2003年工作任务

2003年是全面贯彻落实党的十六大精神的开局年，也是我市建市二十周年的喜庆年。做好今年的政府工作，对于全面推进我市富裕型小康城市建设，具有十分重要的意义。在市第四次党代会上，市委从鄂州实际出发，提出了提前五年实现国内生产总值翻两番、建设富裕型小康城市的目标。按照这一目标，今年政府工作的总体要求是：以邓小平理论和“三个代表”重要思想为指导，认真贯彻落实党的十六大和十届全国人大第一次会议精神，进一步解放思想，实事求是，与时俱进，开拓创新，坚持把发展作为执政兴市的第一要务，全面实施以工业化带动产业化、城镇化和现代化战略，大力推进制度创新、结构创新、科教创新和环境创新，提高经济运行质量和效益，促进物质文明、政治文明和精神文明协调发展，加快富裕型小康城市建设步伐。2003年全市经济和社会发展的预期目标是：国内生产总值比上年增长10%，财政收入增长10%，全社会固定资产投资增长10%，社会消费品零售总额增长10%，外贸出口总额增长10%，实际利用外资增长10%，城镇居民人均可支配收入增长5%，农民人平纯收入增长5%，净增就业岗位1.57万个，城镇登记失业率控制在4%以内，人口自然增长率控制在5‰以内。

完成上述目标，虽然有很多困难要克服，有许多矛盾要解决，但是，我们有很多新的机遇和有利条件，从区位条件上看，鄂州毗邻大武汉，省委、省政府制定的“加快构筑以武汉为中心的城市圈和经济协作区”新方略，为我市接受武汉的辐射和带动，“依武而昌”、加快经济发展，提供了新的契机；从发展基础上看，建市二十年来的持续发展，全市经济结构调整步伐加快，支柱产业逐步形成，经济实力大为增强，为新一轮经济大发展奠定了坚实基础；从发展环境上看，通过深入开展“三个代表”学教和“学查改”活动，经济发展环境大为改善，进一步坚定了投资者来我市投资创业的信心；从发展动力上看，国企“两改”基本完成，招商引资不断扩大，特别是党的十六大和全国“两会”的胜利召开，极大地鼓舞了广大干部群众的斗志，全市上下政通人和、精神振奋，为加快发展提供了强大动力。我们一定要坚持把发展作为执政兴市第一要务，聚精会神搞建设，一心一意谋发展，与强的比，向高的攀，同勇的争，跟自己的潜能挑战，实现经济总量大增长，开放型经济大突破，民营经济大发展，农业产业化经营大提高，第三产业大繁荣，城市面貌大改观。只要我们振奋精神，真抓实干，就一定能够完成和超额完成今年的各项任务。

 (一)实施“一化带三化”战略，统筹城乡经济社会发展  实现建设富裕型小康城市的宏伟目标，关键在城乡协调，全面发展。实施以工业化带动产业化、城镇化、现代化的战略，可以更好地统筹城乡经济社会发展，实现工业与农业，城市与乡村的良性互动。因此，今年乃至今后一个相当长的时期，我们必须把“一化带三化”作为加快建设富裕型小康城市一项重要战略措施来抓。

大力发展工业经济。工业是立市之本、强市之基，是推进城镇化的基础，加快产业化的前提，实现现代化的必由之路。要把工业的发展摆在更加突出的位置，作为经济工作的“牛鼻子”来抓，今年要在四个方面有新突破。一是在发展高新技术产业上要有新突破。以葛店高新区为依托，抓住建设国家级生物技术与新医药产业基地的机遇，着力打造“中国药谷”，重点抓好趋势科技、科益药业、春天生物、人福科技二期等生物医药项目的开发建设，尽快形成生物技术与新医药产业的优势和特色；以电脑磁盘、非接触式IC卡读写终端等为重点，建设与武汉“中国光谷”对接的电子信息产业；完成阻隔功能材料、纳米晶化增强剂的中试，扩大PTC功能陶瓷、特种合金焊接材料和纳米非金属材料的生产规模，加快新材料产业的发展步伐。二是在现有骨干企业的技改上要有新突破。要利用高新技术和先进适用技术改造提升冶金、服装、建材等传统产业，加大骨干企业技改力度，确保全年完成9亿元(全口径)技改投资任务。支持鄂钢、多佳、武昌鱼、顾地塑胶、富特波尔、科氏沥青、鄂城水泥厂、枫树集团等企业做大做强，出精品，创名牌，提高企业核心竞争力和产品市场占有率。同时要高度重视科技含量高、市场潜力大的特色产品的开发，走“小而专”、“小而特”、“小而强”的路子，重点扶持发展燕矶金刚石刀具系列产品，使燕矶逐步发展成为享誉全国的金刚石刀具生产基地和专业市场。三是在发展园区经济上要有新突破。把经济园区作为加快工业化的载体和聚集区，在重点抓好葛店高新区发展的同时，搞好樊口、小桥、新庙、花湖等工业园区的开发和建设，充分运用市场手段，加强园区的基础设施建设，赋予园区相应的经济自主权和项目审批权，增强园区活力，制订优惠政策，引导和促进工业向园区集中，形成规模效应和集聚效应，以局部性跨越式发展带动经济整体跨越发展。四是在项目建设上要有新突破。项目是科技和投资的载体，也是工业发展的重要支撑。发展工业经济，就是要抓现的、上快的，瞄准后劲大的。要以市场为导向，发扬“想尽千方百计，走遍千山万水，说尽千言万语，历尽千辛万苦”的“四千”精神，积极主动地利用境外资本、国家资本、金融资本、法人资本、民间资本，强力吸引项目落户鄂州，做大经济规模，增强发展后劲。全力支持鄂钢进行技改扩张，使其尽快形成年产300万吨钢的规模。大力支持程潮铁矿120万吨球团矿项目建设，确保今年顺利投产；认真做好武钢总规模600万吨球团矿项目的协调服务工作，力促一期工程今年7月动工兴建；抓紧鄂州电厂二期工程前期准备工作，加快多品种化纤项目建设，力促武昌鱼集团城东新区商贸城早日动工建设。

大力推进农业产业化经营。实现全面小康，重点在农村，难点也在农村。要按照“城郊型”农业的定位，围绕农业增效、农民增收的目标，继续调整农业结构，发展优质、高产、高效、生态、安全农业，加快农村小康建设步伐。坚持用现代工业理念谋划农业发展，培育四大支柱产业。按照依法、自愿、有偿的原则进行土地承包经营权流转，使有限的土地资源逐渐向支柱产业、种养大户、技术能手集中，努力形成一乡一品、数乡一业的特色农业板块。水产种养业围绕“精养”做文章，重点建设10万亩名优水产品基地，逐步形成5条10公里的名优水产养殖带。蔬菜瓜果业以“绿色”为标准，重点建设8个万亩无公害蔬菜基地。畜禽养殖业按照生猪调“优”、禽类调“土”的要求，重点建设10个万头“三元猪”、“五元猪”优质商品猪生产基地、20个万只家禽生产基地。花卉苗木业以追求生态效益、美化城乡环境、面向市场需求为目标，重点建设2万亩花卉苗木基地，实施退耕还林和长江防护林工程，完成5万亩造林任务。充分发挥武昌鱼等龙头企业的带动作用，完善“公司+基地+农户”的利益机制，发展各种形式的专业化生产和流通组织，把千家万户的小生产与千变万化的大市场连接起来。引导实业家和经营大户以入股、合伙方式组建农业企业，用订单合同的方式带动农民发展特色种养业。积极发展农副产品加工业，促进农产品多环节转化增值，加粗拉长产业链条。着力抓好特色农产品批发市场建设，积极培育、发展经纪人和行业协会，千方百计搞活农产品流通。加强农产品的监测检验标准体系建设，确保农产品的质量卫生安全，提高农产品的市场竞争力。加强农业科技推广体系建设，搞好农民适用技术培训，办好杜山农业科技示范园。用减少农民的办法富裕农民，有序引导农民外出务工经商，发展打工经济。认真贯彻落实中央和省委、省政府“多予、少取、放活”的方针和关于减轻农民负担的一系列规定，巩固税费改革成果，确保农民负担不出现反弹。抓好长江干堤整险加固、病险水库整治，高度重视抗旱排涝设施建设，加快实施花马湖泵站更新改造工程。加大气象遥感遥测技术服务力度，加快农业机械化步伐，提高农业抗灾避灾能力。

大力推进城镇化进程。围绕“兴山水园林城、建生态旅游市”的目标，加大城市基础设施建设力度。坚持高起点规划、高标准建设、高水平管理、高效能经营的要求，突出抓好城东新区、滨江岸线整治、洋澜湖生态治理、濠塘仿古街、寒溪公园五大工程建设，同时，抓紧做好樊口大桥、华容火车客货运站、鄂黄大桥接线立交桥建设的前期准备工作。城东新区建设是塑造鄂州城市新形象的重大工程，必须把握新型工贸园区的定位，配套搞好公、铁、水交通设施建设，抓住球团矿厂建设的契机，尽快形成新的工业中心和商贸物流中心。坚持“全面规划、依次推进、突出特色、强化功能”的方针，加快小城镇和中心村建设步伐。重点抓好沼山、葛店、燕矶、泽林、庙岭等省级重点镇和华容、太和两个区政府所在镇，以及三山、池湖、三浃、泥矶等省级中心示范村的建设。要树立经营城市的理念，把城镇的建设和管理推向市场。政府垄断土地一级市场，放开放活土地二级市场，全面实行土地使用权公开竞价拍卖。通过拍卖城市土地使用权、桥涵道路冠名权、道路营运权等多种方式筹集城市建设资金，加快城市建设步伐。继续执行对农村村级公路硬化“以奖代补”政策，加速重要经济干线和通乡油路路基建设。加快农村水网、电网、路网、信息网建设，着力改善农村的生产生活条件。以户籍制度改革为突破口，以提高劳动者的素质为重点，促进农村剩余劳动力向非农产业转移，加快城镇化进程。巩固创建中国优秀旅游城市成果，深入开展创建全国文明城、卫生城、园林城活动，提高城市品位。坚持建管并重，加大对城乡结合部、城市道路、街道两侧出店经营、违法建筑的整治力度，完成城市设标工作。坚持可持续发展，认真贯彻落实《环境影响评价法》，不断强化建设项目环境管理，从源头上遏制污染源的产生，促进生态环境的良性循环。进一步提高市民的环保意识，全面启动全国生态市创建工作，努力营造“碧水、蓝天、青山、绿地”的美好环境。加快红莲湖、梁子湖的开发步伐，抓好旅游线路和景区景点建设，形成一批具有地方特色的旅游精品。加强“景区创A、饭店创星、导游创佳”工作，加大旅游商品开发力度，形成旅游产业链。适应大中城市居民休闲、度假的需要，大力发展“农家乐”、“渔家乐”等特色旅游。整顿规范旅游市场秩序，加快旅游人才队伍建设，提高接待水平和服务质量。围绕发展以商贸服务为重点的第三产业，进一步增强城市的流通功能。全面推进连锁经营、物流配送、电子商务等现代流通业的发展。加快生产资料市场、商品市场和要素市场建设，形成各具特色、功能齐全、开放畅通的市场体系。全市人民要进一步解放思想、更新观念，通过大力推进新型工业化、农业产业化、农村城镇化，一步一步迈向现代化。

(二)进一步深化改革、扩大开放，为经济发展注入新的活力 加速富裕型小康城市建设步伐，必须坚定不移地推进各项改革，以更加积极的姿态扩大对外开放，走用改革开放的办法加快经济发展的路子。全面完成国企“两改”。今年，国有企业要全面完成改制转轨。在深化改革中，要坚决冲破一切不利于改革的思想障碍和条条框框障碍，用“三个有利于”的标准来评判是非得失，用“三个有利于”的标准解决改革操作过程中的难点和矛盾，对资产评估等收费、老企业的历欠、银行呆滞贷款、富余职工安置等问题，都要按有利于搞活、有利于发展的原则和实际情况给予特事特办、实事实办。各级各部门各单位特别是各级领导干部，都要勇于为改革与发展承担责任和风险。已完成改制任务的企业，必须认真做好规范完善工作，健全法人治理结构，转变经营机制，加强企业管理，推进股权多元化，巩固和发展改制成果。深化和完善国有资产管理体制改革，逐步建立权利、义务、责任相统一，管资产和管人、管事相结合的国有资产管理体制。突破性发展民营经济。民营经济机制灵活、适应性强、涉及领域宽，是加快鄂州经济发展的又一个新的增长极，也是扩大就业、增加税收、维护稳定的重要基础。加快民营经济发展的关键点，一是真正认识民营经济的地位和作用。坚决贯彻“放心、放手、放胆、放宽、放开、放活”的方针，大力引导和鼓励其健康发展。二是为民营经济的发展创造一个公平竞争的环境。做到在思想观念上平等对待；在从业人员、经营范围、经营方式上平等对待；在土地、资金、能源、原材料等生产要素供给上平等对待；在金融服务、提供信贷支持上平等对待；在社会保障体系建设上平等对待；在鼓励做大做强、募股上市、裂变扩张政策上平等对待；在政治待遇上平等对待。三是培养、吸引、集聚民营经济的带头人和创业者。各项事业的发展靠人，同样，民营经济的发展也要靠一大批敢闯市场、敢冒风险、敢为天下先的经商办实业的能人。各级领导要主动与民营经济带头人交朋友，多理解他们的甘苦，多了解他们发展过程中的问题和想法，及时协调解决他们发展过程中遇到的矛盾和问题，做到以事业留人、以感情留人、以发展机会留人，使企业主安心创业、守法经营、裂变发展。要淡化“铁饭碗”和“官本位”意识，鼓励更多的人进入自主创业的行列。四是积极引导民营企业向科技型、外向型转变，向园区集中，集约发展。进一步扩大对外开放。加快外向型经济发展是适应经济全球化的必然要求，是迅速提升一个地区参与国内外竞争能力的重要途径，是培育高新技术产业的重要手段，是扩大就业、增加税源的重要举措，也是一个地区加快经济发展、做大经济规模、增强经济实力的现实选择。实现开放型经济特别是利用外资的新突破，我们必须贯彻“六个并举”，落实“四项硬措施”。“六个并举”就是扩大开放必须大、中、小并举，独资、合资、嫁接改造并举，一、二、三产业并举，市、区(街办)、重点乡镇、园区并举，外资、外经、外贸并举，内联外引并举，形成全面开放、全员招商的浓厚氛围。“四项硬措施”：一是要有一套灵敏高效的情报信息系统。扩大招商引资要知“四情”，即上情、下情、内情、外情。尤其要及时了解外商的投资意向，包括投资项目、目标、条件、方式等，先人一步、主动跟踪，使招商有对象、有目标、有内容、有准备，做到有的放矢，务求实效。建立“项目库”，实行资源共享，随用随调。二是要有一套行之有效的招商办法。采取“走出去”与“请进来”相结合、大团组与小分队相结合等方式，以企引外，以优引外，以诚引外，以外引外，努力提高招商引资的实际效果。要注重依托武汉城市圈的优势，吸引资金、技术、人才向我市聚集，合理调整产业布局，推进经济一体化进程。三是要有一套灵活、开明的优惠政策和激励措施。根据新的形势和发展开放型经济的迫切需要，对有关政策加以补充完善，形成一套可操作、能兑现的外商投资优惠政策和引进外资有功人员奖励政策。政策一经出台，一定要坚决兑现，绝不能失信于外商，失信于民。四是要有一个高效、规范、诚信、安全、文明的投资环境。要以市区和葛店开发区为重点，实行一个窗口对外、一条龙服务、一个星期内办好手续的制度，并争取建立“零收费”区。总之，对外开放、招商引资要做到诚信到位，效率到点，措施到底。要以外贸出口为重点，不断提高我市经济的外向度。积极开拓国际市场，扶持鼓励企业创造条件自营出口，大力调整出口产业结构和产品结构，增加具有比较优势的服装、农副产品的出口数量，进一步扩大机电产品、高新技术产品的出口规模，力争外贸出口海关数达到5950万美元。大力发展国际经济技术合作，努力扩大劳务输出。切实做好社会保障工作。进一步加强城镇职工养老、失业、医疗等保险制度建设，完善社会保障体系。抓好“两个确保”和“三条保障线”的落实，确保国有企业下岗职工基本生活费和离退休人员基本养老金按时足额发放；落实好城市居民最低生活保障制度，继续把符合条件的城镇贫困人口全部纳入最低生活保障范围，做到应保尽保。扩大社会保险覆盖面，依法做好民营企业和困难群体的参保工作。认真落实中央、省关于下岗职工再就业的一系列优惠政策，大力发展就业容量大的劳动密集型产业，积极开发公益性岗位，组织劳务输出，加强劳动力市场建设，努力为下岗失业人员提供更多的就业岗位。积极引导下岗失业人员转变就业观念，鼓励自谋职业、自主创业和多种形式就业。加强劳动监察力度，保护劳动者的合法权益。关心社会弱势群体，加大扶贫力度，认真组织好部门、单位对口扶贫，动员社会各界参与扶贫，高度重视重点贫困乡镇和老区建设，改善贫困群众的生产生活条件。

积极构建公共财政体制。大力加强财源建设，市直(含葛店开发区)今年要有10个税收过千万元的企业，一个过亿元，一个过3亿元的企业，努力增加财政收入。加大区、乡镇(街办)财源建设力度，不断培植新的财源。鄂城、华容、梁子湖三个区都要培植1—2个税收过千万元的企业，重点乡镇和四个街办都要培植3—5个税收过百万元的企业。今年要有7个乡镇街办财政收入过千万元。加强对增值税、营业税、所得税等主要税种的征管，加大对欠税大户的清缴力度，确保应征税额及时足额入库。坚持量入为出，以收定支，进一步健全和完善零户统管、部门预算、政府采购、国库集中收付、财会集中核算和收支两条线的财政管理体制，优化财政支出结构，加大对基础性、公益性重点项目的支出，逐步使财政资金退出一般性竞争领域，加速由吃饭型财政向建设型财政转变。强化社会信用意识，大力实施信用工程。积极协调银企关系，鼓励银行支持有效益、守信用企业的发展。认真落实对中小企业，特别是民营企业、农业和农民的信贷支持措施，发展个人消费信贷。深入开展“农村信用工程”建设，引导农村信用社调整信贷结构，扩大农村小额贷款的覆盖面和规模，促进农村经济发展。

(三)实施科教兴市战略，促进社会全面进步 坚持科教创新， 致力于建设学习型城市，促进人的全面发展，不断提高科技对经济增长的贡献率，努力推进经济和社会协调发展。

大力发展教育事业。坚持教育优先发展，继续贯彻“一保、二控、三监管”的工作方针，完善基础教育“以区为主”管理体制。全面推进素质教育，实施新课程改革，巩固普及九年义务教育成果，加快高中教育发展步伐，严格控制中小学生辍学，坚决拆除D级危房，改造C级危房，确保师生的生命安全。加大教育资源的整合力度，加快鄂州职业大学建设步伐，为办成本科院校创造条件。深化教育综合改革，加强和改进学校德育工作，健全考试管理制度；大力发展职业教育、成人教育、民办教育，建立终身教育体系；继续实施“教师素质工程”，不断完善教育人事制度改革工作。进一步提高教育现代化、信息化水平，建成开通“鄂州教育信息网”，发展现代远程教育，加快推进教育产业化进程。加大科技创新力度。围绕产业升级，推进科技开发、技术引进和成果转化，重点抓好国家“十五”重大科技专项“双低油菜”优质高效技术研究与示范等一批实用技术的推广。发挥企业在技术创新中的主体作用，加强与高等院校、科研院所的紧密合作，建设企业工程技术中心，努力增强企业的核心竞争力。大力实施制造业信息化工程，以省级示范企业鄂丰模具公司为重点，扎实推进7家市级示范企业的信息化工程建设。进一步加强引智工作，聘请外国专家来我市解决关键技术性难题。加快推进科技资本与金融资本、产业资本的融合，加强技术市场及中介服务体系建设，不断提高科技对经济增长的贡献率。坚持以人为本，鼓励技术、管理等要素参与分配，建立健全有利于留住人才、人尽其才的激励机制和工作环境，努力建设一支适应市场经济的高素质人才队伍。

加强精神文明建设。继续深入贯彻《公民道德建设实施纲要》，在全社会倡导“爱国守法、明礼诚信、团结友善、勤俭自强、敬业奉献”的基本道德规范。广泛开展文明市民、文明单位、文明行业、文明社区、文明镇(村)等多种形式的创建活动，注重总结推广精神文明建设先进经验，不断提高全社会的文明程度。大力弘扬先进文化，进一步繁荣文化艺术、广播电视、新闻出版事业。深化文化体制改革，鼓励和引导文化艺术团体走产业化、市场化、规范化的发展道路。继续开展“扫黄”、“打非”活动，整顿和规范文化市场。加强科普宣传教育，破除封建迷信，倡导科学精神和文明健康的生活方式。

切实维护社会稳定。正确处理新形势下人民内部矛盾，积极稳妥地处理好群体性事件，完善信访日接待制度，倾听群众呼声，解决群众困难。强化社会治安综合治理，确保各种不安定的因素和事件苗头不升级、不扩大、不恶化，超前防范、打击敌对势力的渗透破坏，严厉打击各种违法犯罪活动，严防“法轮功”邪教组织反弹，切实维护社会稳定。深入开展普法教育，增强全体公民的法制观念。强化安全生产意识和责任制，落实各项劳动保护措施，杜绝特大事故，严格控制事故率。继续做好市场经济秩序整顿工作，坚决打击各种制假售假、敲诈勒索、欺行霸市的违法行为。加快发展各项社会事业。加大卫生体制改革力度，完善农村医疗服务网络，全面实施农村改水改厕工程，改善农民群众的生活条件；坚持预防为主方针，加强公共卫生监督，努力提高人民群众的健康水平。加快体育基础设施建设，广泛开展群众体育健身活动，有重点发展竞技体育。继续加强人口和计划生育工作，切实做好农村人口、流动人口和下岗失业人员的计划生育管理和服务，稳定低生育水平。加强社区建设，积极探索新的社区服务运作模式，不断扩大社区服务领域。加强全民国防教育，抓好城市民兵工作调整改革，深入开展“双拥”活动。做好民族宗教、对台、外事侨务、人防、物价、统计工作。维护妇女、儿童的合法权益，关心老龄人和残疾人生活。 为人民群众办实事，是贯彻“三个代表”重要思想的具体体现，是各级政府应尽的职责。今年，市政府在认真抓好经济建设和社会发展各项工作的同时，将集中力量办好六件实事：(1)实施农村改水工程，基本实现乡乡镇镇通自来水，村村户户饮用清洁卫生水；(2)实现村村通水泥路，构建便捷的农村交通网络；(3)启动天然气工程，提高市民生活质量；(4)继续实施洋澜湖综合治理，优化城市生态环境；(5)兴建凤山垃圾处理场，改善居民卫生条件；(6)建立法律援助机制，为社会弱势群体提供法律服务。

三、切实转变职能，打造“服务型”政府做好今年的各项工作，必须按照“廉洁、勤政、务实、高效”的要求，切实转变政府职能，实现由“全能政府”向“有限政府”转变，由“管理型政府”向“服务型政府”转变，不断加强政治文明建设，优化经济和社会发展环境。加快转变政府职能。适应加入WTO和发展市场经济的要求，切实把政府职能转变到经济调节、市场监管、社会管理和公共服务上来，坚持按经济规律办事，用改革的措施、市场的办法来推进各项工作。加大行政管理体制改革力度，按照统一部署，进一步调整机构，理顺职能。加快行政审批制度改革步伐，继续清理与世贸组织规则不相符合，与市场经济体制不相适应和经实践证明有碍经济发展的各类政策、文件，简化保留审批事项的审批程序，实行行政审批限时服务，尽快建立行之有效的审批监督约束机制和责任追究制度。继续全方位地推进政务公开，特别是对涉及人民群众切身利益的部门职能、办事规则、收费依据、处罚标准，必须通过各种方式真实、全面地告知社会，不断提高政府工作的透明度。进一步加强市经济发展联合办公中心建设，扩大“一站式”服务范围，使之尽快成为“政务超市”，切实为经济主体提供优质服务，真正做到下放一切可以下放的权力，取消一切可以取消的收费，简化一切可以简化的手续。按照政事分开、分类管理的要求，切实做好撤销、合并、改企、保留事业单位的分类改革，全面完成市直事业单位人事代理制及未聘人员分流安置工作，努力在事业单位建立起单位自主用人、人员自主择业、职务能上能下、待遇能升能降、优秀人才脱颖而出的用人机制。全面推行依法行政。加强社会主义民主法制建设，改革和完善决策机制，建立与群众利益密切相关的重大事项社会公示制和社会听证制度，完善专家咨询制度，不断推进决策的民主化、科学化。加强政府法制建设，进一步完善行政执法责任制和错案责任追究制，形成以目标考核为核心，以量化责任为标准，以全程监督为手段，以考核奖惩为动力的行政执法管理体制。大力推行行政综合执法，相对集中行政处罚权，切实解决“多头执法”、“多层执法”的突出问题。自觉接受人大及其常委会对政府工作的法律监督，并定期报告工作；自觉接受政协的民主监督，完善民主协商机制；支持民主党派参政议政，认真办理人大代表议案、建议和政协委员提案。努力改进工作作风。按照务必继续地保持谦虚谨慎、不骄不躁的作风，务必继续地保持艰苦奋斗的作风要求，坚决压缩会议费、招待费、出国考察费，严格控制新建办公楼、购置小汽车，机关宿舍建设一律按市场方式运作。注重深入基层，深入群众，加强调查研究，坚持说实话、报实数、办实事、求实效，精简会议文件，减少应酬接待，坚决反对官僚主义和形式主义。大兴学习之风，加强学习型机关建设，不断提高政府工作人员的整体素质。加快以“电子政务”为重点的政府管理信息化建设，推进政府工作方式现代化。进一步强化工作目标责任制，加大年度工作目标、重点工作和重点项目的督查落实力度，健全责任分解、考核和追究的工作体系，不断提高行政效率。加强对政府机关工作人员的严格要求、严格教育、严格管理、严格监督，强化审计、监察职能，以“制权、管钱、用人、公开”为重点，加大源头治腐力度，树立清正廉洁的政府形象。

切实优化发展环境。坚持以“三个代表”重要思想为指导，牢固树立抓环境就是抓发展、以经济建设为中心就是以企业为中心的思想，满腔热情地为企业服务，积极主动地为企业排忧解难，对企业要慎用罚款，对企业法人要慎用强制措施，对企业生产经营出现的一些问题要正确引导，加强教育，促其规范管理，依法经营，健康发展。坚持以思想教育和加强监督为重点，增强全体市民尤其是政府工作人员的环境意识和服务观念；以规范收费行为、依法整治“三乱”为重点，坚决制止各种侵害经济主体和人民群众利益的不正之风，斩断伸向企业的“黑手”；以企业周边、学校周边、交通沿线、建筑工地、集贸市场为重点，严厉打击各种扰乱社会秩序和企业正常生产经营的违法犯罪行为，努力营造公正严明的法制环境、规范有序的市场环境、诚实守信的信用环境、优质高效的服务环境及文明和谐的人文环境。各位代表，今年是本届政府任期的最后一年，加快鄂州改革开放和经济社会发展的任务十分艰巨。让我们紧密团结在以胡锦涛同志为总书记的党中央周围，在党的十六大精神指引下，在省委、省政府和市委的正确领导下，高举邓小平理论伟大旗帜，全面贯彻“三个代表”重要思想，与时俱进，开拓创新，团结拼搏，扎实工作，圆满完成本次会议提出的各项任务，以优异成绩迎接建市二十周年，为把我市建设成为富裕型小康城市而努力奋斗！
